# Cardiac involvement in a patient with clinical and serological evidence of African tick-bite fever

**DOI:** 10.1186/1471-2334-5-90

**Published:** 2005-10-20

**Authors:** Cristina Bellini, Matteo Monti, Mathieu Potin, Anne Dalle Ave, Jacques Bille, Gilbert Greub

**Affiliations:** 1 Infectious Diseases Unit, University Hospital of Lausanne, Lausanne, Switzerland; 2 Internal Medicine Unit, University Hospital of Lausanne, Lausanne, Switzerland; 3 Center for Research on Intracellular Bacteria, Institute of Microbiology, University of Lausanne, Lausanne, Switzerland

## Abstract

**Background:**

Myocarditis and pericarditis are rare complications of rickettsiosis, usually associated with *Rickettsia rickettsii *and *R. conorii*. African tick-bite fever (ATBF) is generally considered as a benign disease and no cases of myocardial involvement due to *Rickettsia africae*, the agent of ATBF, have yet been described.

**Case presentation:**

The patient, that travelled in an endemic area, presented typical inoculation eschars, and a seroconversion against *R. africae*, was admitted for chest pains and increased cardiac enzymes in the context of an acute myocarditis.

**Conclusion:**

Our findings suggest that ATBF, that usually presents a benign course, may be complicated by an acute myocarditis.

## Background

Myocarditis and pericarditis are rare complications of rickettsiosis, usually occuring in the setting of an acute disseminated infection due to *Rickettsia rickettsii *and *R. conorii *[1-3].

*Rickettsia africae*, the causative agent of African tick-bite fever, an emerging disease transmitted by Amblyomma ticks in rural sub-Saharan Africa, has been recently described [4]. Symptoms usually includes abrupt appearance of fever (59–100% of cases), headache (62–83%), myalgia (63–87%), prominent neck muscle myalgia (81%), regional lymphadenitis (43–100%), cutaneous rash (15–46%) and inoculation eschar (53–100%), typically present in multiple sites (21–54%) [1]. The time lag from tick bite to symptom onset is usually 5 to 7 days but may be as long as 10 days [2-4].

Several case reports of ATBF in travellers from Europe and elsewhere have been published [1-5] and recently, ATBF have also been reported in autochthonous Africans [6]. However, no cases of myocardial involvement have yet been described. Here, we report the first evidence that ATBF may be complicated by an acute myocarditis.

## Case presentation

**Figure 1:**
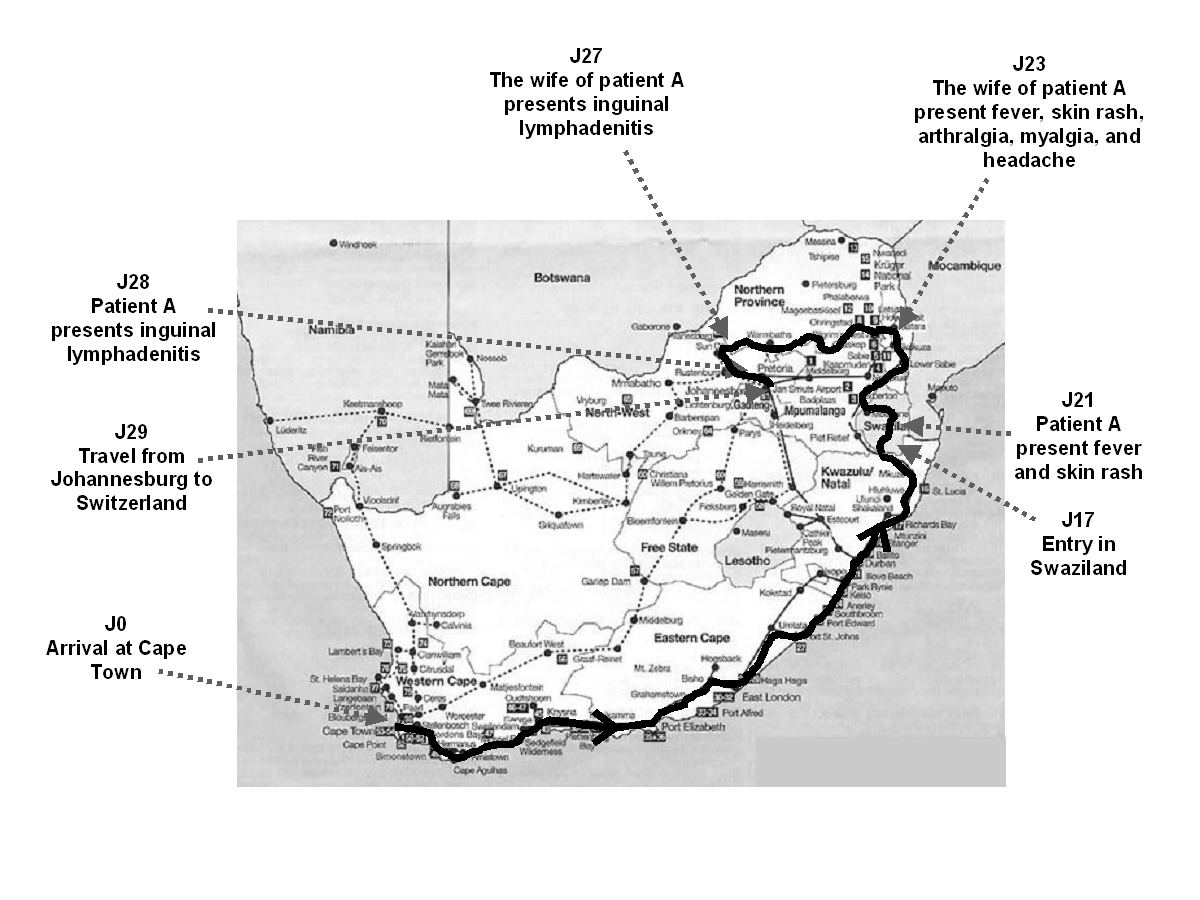
Travel of patient A and his wife in South Africa and Swaziland and description of key events, signs and symptoms.

Patient A and his wife, two healthy 35 years old adults, did a 4-weeks camping's holidays in South Africa in July 2004. They travelled along the southern coast (Figure 1), and were daily exposed to insect and tick bites. Four days after their arrival in Swaziland and 19 days after the beginning of the trip, patient A suddenly presented fever and a vesicular erythematous rash located on both legs. His condition improved with acetylsalicylic acid. However, he developed in the following week several inguinal lymphadenitis and severe asthenia. His wife presented – with a two days delay – an acute febrile illness, associated with a macular rash on both legs, myalgias, arthralgias and headache. Her fever resolved within 24 hours. However, skin lesions were still present four days later, when she observed a new inguinal lymphadenopathy. Seven days after the symptoms onset, they returned to Switzerland. Two days later, patient A presented an oppressive chest pain and profuse perspiration, which spontaneously resolved in 3 hours. Because of this acute episode, he went to the emergency unit of Lausanne's University hospital.

**Figure 2:**
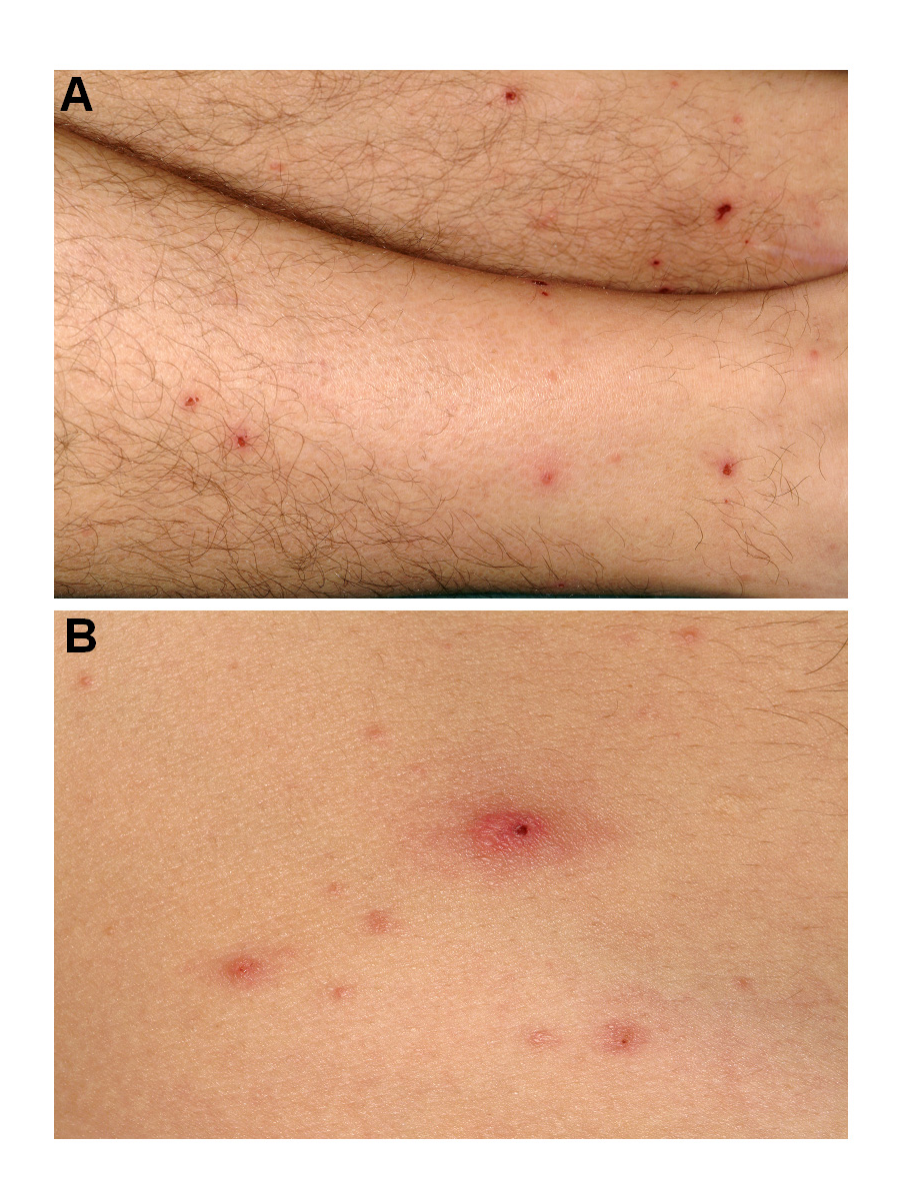
Skin lesions (patient A): 2A. vesicular erythematous rash of both legs; 2B. one inoculation eschar on the abdomen.

**Figure 3:**
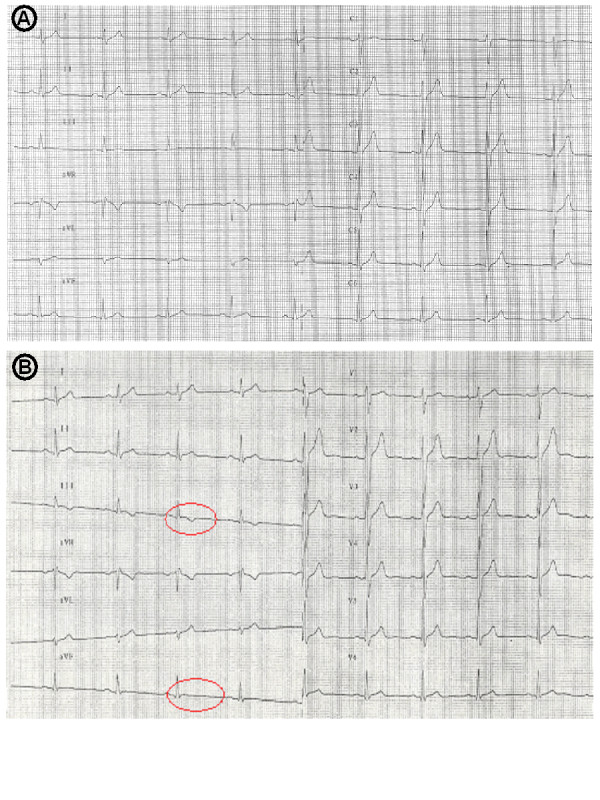
Electrocardiograms performed (A) at admission and (B) 6 hours later: repolarisation abnormalities not present initially are present in the inferior leads of the 2^nd ^electrocardiogram.

At admission, all symptoms including chest pain, were resolved. He was afebrile. Multiple reddish vesicles, partially crusted, measuring 1 to 2 mm, were present on the legs and on the abdomen (Figure 2A). We also observed a 10 mm infiltrated abdominal lesion with a small central necrosis similar to typical tick-bite inoculation eschars (Figure 2B). There were no signs of heart failure and cardiac auscultation was physiological, without friction rub. To investigate the thoracic pain, an electrocardiogram was performed, which was normal, without signs of ischemic heart disease. Cardiac enzymes were slightly increased, with CK, CK-MB, and troponin I level in blood of 330 UI/l (Normal Values [NV] 25–190 UI/l), 40% (NV <6%), and 2,12 mg/ml (NV < 0.04 mg/ml), respectively. The other blood analyses revealed a moderate leucopenia (3.7 G/l, NV 4–10 G/l), and a slight increase of hepatic enzymes. A normal thoraco-abdominal Computer Tomography excluded aortic dissection. About 6 hours after the first electrocardiogram, repolarisation abnormalities were observed in the inferior leads (Figure 3). This was associated with an increase of cardiac enzymes (peak of CK and Troponin I of 400 UI/l and 3.62 mg/ml respectively). A coronarography showed normal vessels. Both cardiac echocardiographies performed respectively at admission and two weeks later, showed no pericardial effusion and a normal ejection fraction of about 65%. A rickettsial disease was suspected based on the presence of a febrile illness with a rash and inoculation eschars. The history of multiples tick bites and the simultaneous occurrence of the disease in both patients were suggestive of ATBF. This diagnosis was confirmed by a *R. africae *seroconversion of patient A (titres of 1/64 in IgG and 1/16 in IgM on the convalescent serum taken one month after the symptom onset) and by a sustained positive *R. africae *serology for his wife (IgG titers of 1:64 and IgM titres of 1:32 on both acute and convalescent sera; IgG titers >= 1:64 and IgM titers >= 1:32 are considered indicative of infection by *R. africae*). As frequently observed, similar antibody titres were obtained for other spotted fever group rickettsia, such as *R.conorii *and *R. massiliae*. Western-blot and cross-adsorption that may help to precise the etiological agent [11] was not feasible due to the low antibody titres. Both patients were treated with doxycycline 200 mg daily with rapid recovery.

## Conclusion

Contrarily to *R. rickettsii *and *R. conorii *that are considered potential agents of myocarditis and pericarditis [1-3], *R. africae *usually present a benign, uncomplicated course, and has never been yet associated with cardiac complications. Our findings suggest that *R. africae*, the agent of ATBF, may lead to myocarditis.

In this report, the grouped cases, the presence of multiple inoculation eschars and the serologic seroconversion for *R. africae*, strongly supported the diagnosis of ATBF. To confirm the diagnosis, we could also perform PCR and/or culture on the biopsy of the eschar bite, since both techniques are good tools to diagnose acute rickettsial infection [4]. However, no skin biopsy was performed for ethical reasons since the diagnosis of ATBF was evident.

In presence of highly specific pathologic cardiac enzymes (troponin I) and ECG alteration, and in absence of coronary stenosis at coronarography, and despite the absence of morphological abnormalities at echocardiography, the more likely cause of the chest pain is a myocarditis with or without pericardial involvement. Indeed, myocarditis that is defined as an acute inflammatory syndrome involving the heart and related structures is typically characterized by increased troponin and normal coronary arteries [13-15]. Since the pathogenesis of rickettsial disease is generally associated with endothelium damage [1], unstable angina might occur in patients with spotted fever Rickettsiosis. However, in this case, the normal coronarography does not support the occurrence of a peripheric, transitory thrombotic event. A myocarditis is much more likely in this setting.

To our knowledge, there are no described case of cardiac involvement associated with a *R. africae *infection. Although a serological cross reaction with another rickettsial infection can not be formally excluded, the endemic presence of *R.africae *in Swaziland and South Africa [2-4], the presence of multiple inoculation eschars and the simultaneous infection of both travellers [5], strongly support the diagnosis of ATBF.

In conclusion, if ATBF usually presents a benign course, rare complications such as myocardial involvement may occur. Travellers to endemic areas should be informed of the risk of contracting ATBF and be encouraged to take personal protective measures against tick bites [5,7].

## List of abbreviations

ATBF = african tick bite fever

CK = creatine phosphokinase

CK-MB = creatine phosphokinase muscle-brain isoform

NV = normal value

UI= international unit

PCR = polymerase chain reaction

ECG = electrocardiogram

## Competing interests

The author(s) declare that they have no competing interests.

## Authors' contributions

All authors were involved in patient care. CB wrote the first draft of the paper. All authors improved the manuscript and approved its final version.

## Pre-publication history

The pre-publication history for this paper can be accessed here:


